# The experiences of hospitals in changing the function of a non-teaching hospital to a teaching hospital: Short-Communication

**DOI:** 10.22088/cjim.14.2.365

**Published:** 2023

**Authors:** Niusha Shahidi Sadeghi, Mohammadreza Maleki, Hassan Abolghasem Gorji, Soudabeh Vatankhah, Bahram Mohaghegh

**Affiliations:** 1 Department of Health Services Management, School of Health Management and Information Sciences, Iran University of Medical Sciences, Tehran, Iran; 2 Department of Public Health, School of Health, Qom University of Medical Sciences, Qom, Iran

**Keywords:** Teaching hospitals, Systems Integration, Health Systems Agencies, University hospitals

## Abstract

**Background:**

In recent years, there have been many non-teaching hospitals that have become teaching hospitals. Although the decision to make this change is made at the policy level; But the unknown consequences can create many problems. The present study investigated the experiences of hospitals in changing the function of a non-teaching to a teaching hospital in Iran.

**Methods:**

A Phenomenological qualitative study was conducted using semi-structured interviews with 40 hospital managers and policy makers who had the experience of changing the function of hospitals in Iran through a purposive sampling in 2021. Thematic analysis using inductive approach and MAXQDA 10 was used for data analysis.

**Results:**

According to the results extracted 16 main categories and 91 subcategories. Considering the complexity and instability of command unity, understanding the change of organizational hierarchy, developing a mechanism to cover client’s costs, considering increase management team’ legal and social responsibility, coordinating policy demands with Providing resources, funding the teaching mission, organizing the multiple supervisory organizations, transparent communication between hospital and colleges, understanding the complexity of processes, considering change the performance appraisal system and pay for performance were the solutions for decrease problems of changing the function of non-teaching to teaching hospital.

**Conclusion:**

Important matter about the improvement of university hospitals is evaluating the performance of hospitals to maintain their role as progressive actors in hospital network and also as the main actors of teaching future professional human resources. In fact, in the world, hospital becoming teaching is based on the performance of hospitals.

Hospitals as the main lever of countries health system are divided into two teaching and non-teaching hospitals (1). According to 2017 statistics in Iran, around 45% of the whole active beds and around 63% of Ministry of Health active beds are teaching which is increasing (2). But according to statistics, this rate is much lower in other countries for example only 5% of all US hospitals (3). According to the preface and initial review, it is expected that the difference in the mission of teaching and non-teaching hospitals will cause differences in the outcome indicators (4-10). Teaching hospitals in most countries depend on medical university or a part of national or local health system considering organizational system play a strategic role in teaching physicians (5, 6).

Meanwhile, non-teaching hospitals in most countries are those with general specialty which have activity beside teaching hospitals to protect societies’ health (9). Although the establishment of a hospital should be based on the needs of society and the study of facilities, but the issue of changing medical units, from change of use to change in activity and type of organization, is not a new issue and we have always witnessed this issue in some of countries. The decision to make these changes is made at the policy level; but the unknown consequences and the level of these changes can create problems for everyone. The present study investigated the experiences of hospitals in changing the function of a non-teaching hospital to a teaching hospital in Iran.

## Methods

A Phenomenological qualitative study was conducted in Iran in 2021. The research population consisted of 25 hospital managers at different levels, and 15 policy makers who had the experience of changing the function of hospitals in Iran were recruited through a purposive sampling and by sampling from significant cases. Also, they were from different cities of the country and at least had five years of working experience. Data were collected by using a semi-structured interview to have an in-depth picture of the participants’ perspectives until saturated. An interview guide was prepared based on the research goals, the theoretical foundation of the topic, and an extensive review of the literature (5-11). Interview questions included the following: Are the goals of these two types of hospitals different? What is their goal? Are there other differences between teaching and non-teaching hospital? What were your experiences about these hospitals?

Data were gathered during a 4-month period from April-June 2021. The validity of the data was based on four indicators expressed, including credibility, dependability, confirm ability, and transferability. The interviews lasted from 55 to 120 minutes and were immediately transcribed after each session. 

To analyze data, the inductive thematic analysis approach according to Braun and Clarke was used. MAXQDA was used for data analysis. Accordingly, 1) data coder immersed in the data by listening, reading and re-reading 2) the initial list of ideas behind the data was generated, and the initial codes from the data were produced, 3) the data were coded and then analyzed thoroughly 4) the themes and sub-themes were reviewed and refined with the research team, 5) reviewed final themes were noted considering the cross-links 6) the report was produced. According to ethical considerations, the code of ethics IR.IUMS.REC.1398.259 was obtained from the research deputy of Iran University of Medical Sciences with COI 675856.

## Results

The results of this stage obtained from the analysis of 40 interviews created 354 primary codes, which were minimized to 135 codes after deleting duplicate codes and merging similar codes. Ultimately, the leading codes from the data analysis were assigned in 91 subcategories and 16 categories (table 1). 

**Table 1:** The experience of hospitals in changing the function of a non-teaching hospital to a teaching hospital

**Category**	**sub-category**	**Category**	**sub-category**
** Goals and mission**	Unwavering the principle of unity of purpose Overcoming missions on each other Complexity in prioritizing organizational goals Increasing the need to fit missions with hospital facilities	** Hospital processes**	Increase the complexity of processes Duplication of processes development of new processes Increase dependence in the planning of organizational units on the approvals of committees
**External and Internal communications**	Increase the impact of the fragmented performance of upstream organizations The need for multiple supervisory organizations in issuing licenses The need for a transparent communication process between hospital and related colleges Increase the number and level of contracts Increase in the number and type of people associated with the hospital Increasing the number and level of individual and group communication Change in the type of employment and job description of senior managers Changing the role and position of senior managers Incidence of interpersonal conflict Incidence of intergroup and organizational conflict	**Supply of organizational resources**	Changes in planning and the need for transparency in the funding of the training mission Need to develop training coefficient in hospital budget Need to cover the overhead training costs by the government Change the supply chain management The complexity of the fit of expert human resources and facilities with the multiple missions of the organization Increase hospital influence to provide human resources, equipment and hospital projects Monopoly of some services, manpower and equipment
**Development of clinical and support departments**	Finding the importance of the role and social position of the hospital Strong demand of hospital staff to be the research hospital Increasing the frequency and variety of specialized and sub-specialized wards development of Library unit opportunities of active 24 hours wards developing new committees and more active committees Increasing the frequency and variety of medical equipment	**Cost-effectiveness and efficiency**	Increasing the diversity of foci affecting effectiveness and efficiency Changing the role of senior managers in effectiveness and efficiency Change in the value chain The complexity of coordinating policy demands with the provision of resources for hospital missions The complexity of providing comprehensive arrangements fit to the hospital's missions
**Hospital organizational structure**	Change and increase the scope of manager’s supervision Complexity and instability of command unity Changing the organizational hierarchy Change in the central positions of the organization Need to develop a clear job description for the management team Need to fit the position with the power, authority and accountability of senior managers	**Type and level of services**	Clinical complexity of clients Increasing the frequency and level of outpatient and inpatient services Increasing the contexts of realizing the dream of not dispatching the patient to receive services Upgrading the frequency, type and level of services state tariff for disadvantaged areas Increasing the frequency of paraclinical diagnostic procedures and LOS of the patient Provide faster access to services at night and on holidays
**Internal and external customer satisfaction**	Increasing the diversity of foci’s satisfaction Decreased level of satisfaction Change in the reasons for dissatisfaction Need to inform and clarify missions for internal and external customers Need to develop a mechanism to cover possible material and spiritual costs for clients	**Manpower**	Change in the type and frequency of people in the organization Shift in the use of workforce Changes in guidelines and processes for the role of the workforce in service delivery The need for enhancing the supervision of service delivery with workforce Need to develop a virtual access platform for attends Changing the requirements for empowerment and continuous training of the workforce
**Revenue-cost management**	Increase the hospital's capacity to attract own dedicated revenues Increase the cost of treatment for the patient and the health system Need to restructure pay for performance (training in parallel with medical) Need to develop non-material incentive mechanisms for staff Emergence of hidden costs of education Need to change the way of reimbursement of expenses Increase costs of information and facilities and equipment management	**Performance evaluation**	Change the performance appraisal system components Increase performance indicators Changing performance foci’s response Increasing the need to develop internal incentive regulations Need to develop a comprehensive system of performance evaluation and two sides feedback
**organizational behavior**	incidence of opportunities for changing individual and group behavior at all levels and units opportunities of change in manpower motivation opportunities of higher level of organizational learning Increase the level of need for effective interaction training Providing opportunities to increase staff motivation and organizational commitment Changing the level and methods of individual and group decision making	**Organizing the training mission**	The need to develop an organized structure to ensure the interests of trainees and trainers Need to develop motivational leverage for residents as absorbing elements Need to develop guidelines for respecting the principle of trainee’s respect Need to organize the guidance and education’s supervision of non-medical fields Need to monitor the proper distribution of patients between students
**Empowerment of human capital**	Increase the level of knowledge and skills of staff at all levels Increase motivation and diligence in staff Promotion of personnel by dealing with new issues and people Easier access to training courses	**Legal and social responsibility**	Increase the legal and social responsibility of the management team Increasing the opportunities of Extra-Tariff and guiding the patient to other medical centers Gaining public credit and branding hospitals and specialist physicians Introducing Hospital in the media and news

## Discussion

Most The present study investigated the experiences of hospitals in changing the function of a non-teaching hospital to a teaching hospital in Iran. According to the results of the present study, teaching hospitals are more expensive. Actually, financial and budget resources in these hospitals are mainly pushed to one or two items of tree main functions in various cases (4). The present study findings mentioned the factors which increase the cost of such hospitals. The most important problem in representing health and medical services is its economic problem and hospitals in Iran are still faced with this problem (10). According to the results of the present study, the important point is that in some countries extra resources have been appropriated to university hospitals through specific budget called education, research and or serving complex cases (12). 

Another element of an organization is its structure. According to theory, "structure follows strategy" (13). along with the change in the goal, its structure will also change. Also, according to studies, by changing the purpose of the hospital, many changes occur in human resources (14). This is because building or changing an organization is not like building or changing the layout of a construction (1,14,15). Also, another change is necessary, especially for internal and external communications. It should be noted that the organization is not a simple structure or chart (15).

The next issue to be changed is the "patient" as the main customer of medical institutions (14). The present study emphasizes that more complex cases are referred to teaching hospitals, which is confirmed by the other studies. Medical assistants and interns in medical education system involved in patient medical and care processes may expose the patient to physical, mental and even economic complications (15).

In addition, the results indicate that internal and external customer satisfaction was another category. according to distributive justice although community future interest in training experienced physicians are considered in teaching medicine, it seems that the effect of this matter has not been evaluated on health system (16). 

Based on the results of the present study, the chain of results of teaching and non-teaching hospitals in Iran and the world is different. According to the other studies the output indicators of teaching and non-teaching hospitals are different (4-11).

Organizational behavior has been changed during the change of hospital. in the world, Matrix structure is the helper factor in university medical centers considering their multiple missions (11). In most of world’s university medical centers, the head of clinical unit is accountable to CEO of hospital and also the head of medical university (11). The head of clinical unit is a key connection between the head of university and hospital CEO (12, 17). 

Another issue in this organizational change will be the issue of facility management and in particular the issue of physical space requirements and prerequisites. Facility management in a hospital is the process of reassuring supervisors that a hospital's facilities, equipment and facilities, access, and engineering and architecture support its missions as an important medical institution (18). In the design of a building, it is necessary to study the basic needs related to the list of spaces in relation to the types of functions and its capacity as a physical program.

Finally, Considering the complexity and instability of command unity, understanding the change of organizational hierarchy, developing a mechanism to cover costs for clients, considering increase the legal and social responsibility of the management team, prioritizing organizational goals, coordinating policy demands with Providing resources, funding the teaching mission, organizing the multiple supervisory organizations, transparent communication between hospital and colleges, understanding the complexity of processes, considering the change of individual and group communication, considering change the performance appraisal system and pay for performance were the solutions for decrease problems of changing the function of non-teaching to teaching hospital.

Important matter about the improvement of university hospitals’ performance is evaluating the performance of hospitals to maintain their role as progressive actors in hospital network and also as the main actors of teaching future professional human resources. In fact, for a hospital to become a teaching hospital, its performance is of importance.

**Limitations: **Some experts were not willing to cooperate or participate in the interviews. Some attempts were made to solve this issue and to attract their participation by sending official recommendation letters via our colleagues. As regards the present research was a qualitative study; it was able to study what happens after the change of function in hospitals. However, this can be explored from other perspectives, such as differences in challenges and problems or other study methods can be used.
